# Targeting NRF2 addiction in cancer: synthetic lethal strategies beyond direct inhibition

**DOI:** 10.3389/fcell.2026.1856014

**Published:** 2026-05-21

**Authors:** Melat T. Gebru, David Stokoe

**Affiliations:** Calico Life Sciences LLC, San Francisco, CA, United States

**Keywords:** drug resistance, ferroptosis, glutaminolysis, KEAP1, lung cancer, metabolic reprogramming, Nrf2, reductive stress

## Abstract

Nuclear factor erythroid 2-related factor 2 (NRF2) (encoded by *NFE2L2*) is a master regulator of antioxidant, metabolic, and proteostasis pathways. While protective in normal cells, constitutive NRF2 activation driven by loss-of-function mutations in *KEAP1*, gain-of-function mutations in *NFE2L2*, or non-mutational mechanisms is common in cancer, occurring in approximately 20%–30% of non-small cell lung cancers and at significant frequencies across multiple tumor types. In cancer, the NRF2 transcriptional program drives metabolic reprogramming, drug resistance, ferroptosis evasion, and immune exclusion making these tumors highly therapy resistant. Despite decades of effort, direct pharmacological inhibition of NRF2 has not achieved clinical success due to its structural undruggability, systemic toxicity, and context-dependent biology. This review focuses on targeting NRF2-driven metabolic dependencies as synthetic lethal vulnerabilities, spanning pathways such as glutaminolysis, redox imbalance, cystine metabolism, nucleotide biosynthesis and ER proteostasis. We also highlight emerging strategies, including allosteric KEAP1 activators, and discuss key challenges in translating these approaches into effective therapies.

## 1 Introduction

The maintenance of cellular homeostasis is predicated on a robust response to oxidative and electrophilic stressors, which, if left unchecked, could be detrimental to cells and contribute to a range of human pathologies. Central to this defense mechanism is Nuclear factor erythroid 2-related factor 2 (NRF2), a transcription factor encoded by the *NFE2L2* gene and belonging to the Cap ‘n’ Collar (CNC) subfamily of basic leucine zipper (bZip) proteins. NRF2 serves as a master regulator of over 200 genes involved in antioxidant response, phase II drug metabolism, and metabolic reprogramming, reviewed in (Zhang, 2025).

The discovery of NRF2 originated not from studies of oxidative stress but of globin gene regulation. In 1994, Moi et al. used the tandem NF-E2/AP1 repeat of the β-globin locus control region as a recognition site probe to screen a K562 erythroleukemia cDNA library, with the goal of identifying structurally related CNC-bZIP family members. This yielded a previously uncharacterized 66-kDa protein which they named NRF2 (NF-E2-related factor 2) containing a CNC domain and bZIP region homologous to p45 NF-E2 and NRF1, capable of binding the NF-E2/AP1 motif *in vitro* as a heterodimer with small Maf proteins. Northern blot analysis revealed broad NRF2 expression across non-erythroid tissues, in contrast to the erythroid-restricted pattern of p45 NF-E2, hinting biological roles beyond globin regulation and erythropoiesis (Moi et al., 1994). This was confirmed when *Nrf2* knockout mice, generated by Chan et al. in 1996, were overtly normal and non-anemic, suggesting that NRF2 is dispensable for globin regulation but that its role would potentially only emerge under physiological challenge (Chan et al., 1996). That challenge was identified the following year by Itoh et al. where they showed that phase II detoxifying enzymes including glutathione S-transferase and NQO1 failed to be induced in *Nrf2*-null mice. They showed that NRF2 binds antioxidant response elements (AREs), found in the promoter region of NRF2 target genes, as a heterodimer with small Maf proteins, establishing the NRF2/sMAF-ARE axis as the central transcriptional response to electrophilic stressors (Itoh et al., 1997). The final cornerstone study came in 1999, when the same group identified KEAP1 (Kelch-like ECH-associated protein 1) as NRF2’s principal repressor, showing that it binds the Neh2 domain of NRF2 to suppress its activity under basal conditions and that electrophilic modification of KEAP1 releases this repression to allow nuclear NRF2 accumulation (Itoh et al., 1999). Subsequent work by others established that KEAP1 acts as a substrate adaptor for a CUL3-based E3 ubiquitin ligase, and that the two-site “hinge and latch” interaction between KEAP1 and NRF2 underpins both constitutive NRF2 degradation and its stress-induced stabilization (Cullinan et al., 2004; Kobayashi et al., 2004; McMahon et al., 2006; Tong et al., 2006). In the three decades since these seminal studies, research has revealed that NRF2 functions beyond redox regulation, influencing cellular metabolism, iron homeostasis, proteostasis, and autophagy (Mitsuishi et al., 2012; Hayes and Dinkova-Kostova, 2014; Frias et al., 2020; Anandhan et al., 2023; Zhang, 2025). Crucially, NRF2 dysregulation is a hallmark of various human diseases, including cancer, neurodegeneration, and inflammatory disorders (Rojo de la Vega et al., 2018; Saha et al., 2020; Suzen et al., 2022).

This review examines the role of NRF2 in cancer, its therapeutic relevance and the challenges of targeting it directly. We discuss NRF2 biology in normal cells as well as the oncogenic consequences of constitutive NRF2 activation, including metabolic reprogramming, drug resistance, and tumor microenvironment remodeling. We discuss the current efforts in drugging NRF2 and critically evaluate the challenges of direct inhibition strategies. Finally, we discuss an alternative framework: that the metabolic dependencies imposed by NRF2 addiction in *KEAP1*-mutant tumors represent therapeutically exploitable vulnerabilities. We review an emerging landscape of synthetic lethal strategies and use the selective dependency of *KEAP1*-mutant cells on SLC33A1, UXS1, NADH and ATM kinase as paradigmatic examples of how NRF2’s own transcriptional program can become a liability.

## 2 NRF2 biology and signaling in normal cells

### 2.1 NRF2 protein structural architecture: the Neh domains

**FIGURE 1:**
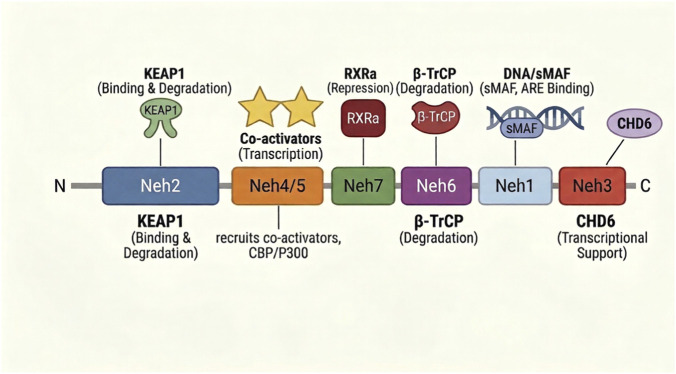
Modular domain architecture of NRF2 and key regulatory interactions. Schematic of the seven NRF2-ECH homology (Neh) domains from N- to C-terminus. Neh2 recruits KEAP1, targeting NRF2 for proteasomal degradation under basal conditions. Neh4/5 recruit co-activators CBP/p300 to drive target gene expression. Neh7 mediates repression via RXRα interaction. Neh6 contains phosphodegrons that recruit β-TrCP for KEAP1-independent degradation. Neh1 harbors the CNC-bZIP motif required for sMAF heterodimerization and ARE binding. Neh3 supports transcriptional activation through CHD6 interaction.

The functional versatility of NRF2 derives from its modular architecture, which comprises seven conserved NRF2–ECH homology (Neh) domains that coordinate protein stability, transcriptional activity and protein–protein interactions (Figure 1).

Primary regulation of NRF2 stability is mediated through the N-terminal Neh2 domain, which serves as the docking platform for KEAP1, discussed in detail in the next section below. A secondary, redox-insensitive degradation pathway is mediated by the Neh6 domain, which contains two serine-rich phosphodegrons (DSGIS and DSAPGS) that, upon GSK-3β-mediated phosphorylation, recruit the E3 adaptor β-TrCP to direct NRF2 for CUL1-based proteasomal degradation independently of KEAP1 (Salazar et al., 2006; Rada et al., 2011; Chowdhry et al., 2013). This pathway is particularly relevant in settings where KEAP1 function is impaired, including *KEAP1*-mutant tumors, as it provides a residual brake on NRF2 activity that may influence the magnitude and duration of target gene induction (Okazaki et al., 2020).

DNA binding and dimerization are mediated by the Neh1 domain, which contains a CNC motif and bZIP region. Neh1 is required for NRF2 to form heterodimers with small Maf proteins (MafF, MafG, MafK), enabling the complex to bind AREs in target gene promoters (Itoh et al., 1997). Transcriptional activation is achieved through the synergistic action of the Neh4 and Neh5 transactivation domains, which recruit the co-activators CBP/p300 and the chromatin remodeler BRG1, while the C-terminal Neh3 domain supports assembly of the transcriptional apparatus through interaction with Chromodomain Helicase DNA-binding protein 6 (CHD6) (Katoh et al., 2001; Nioi et al., 2003; 2005; Zhang et al., 2006). Neh7 domain mediates NRF2 repression through direct interaction with retinoic X receptor alpha (RXRα), competing with sMAF proteins for NRF2 binding in a ligand-independent manner (Wang et al., 2013).

Collectively, the partitioned Neh domain architecture positions NRF2 as a highly regulated signaling node whose activity is fine-tuned by redox state, kinase signaling, and protein-protein interactions, a complexity that, as will be discussed, has significant implications for therapeutic targeting.

### 2.2 The KEAP1-NRF2 regulatory axis

**FIGURE 2:**
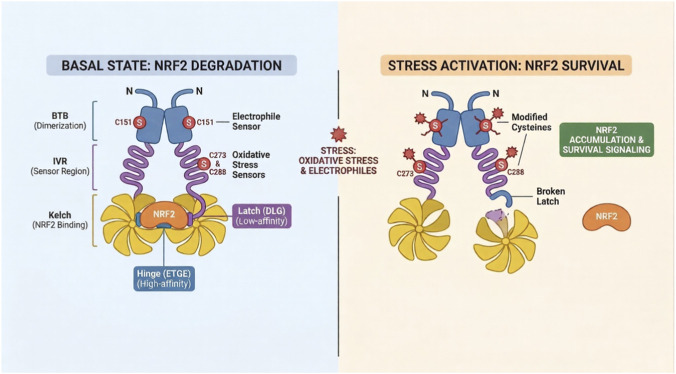
The KEAP1-NRF2 regulatory axis under basal and stress conditions. (Left) Basal state. KEAP1 homodimerizes and engages NRF2 through its Kelch domain at two sites the high-affinity ETGE hinge and lower-affinity DLG latch positioning NRF2 for polyubiquitination by the CUL3-RBX1 E3 ligase and proteasomal degradation. Reactive cysteines C151, C273, and C288 serve as redox-sensitive sensors that regulate this interaction. (Right) Stress activation. Modification of KEAP1 sensor cysteines disrupts the DLG latch, preventing NRF2 ubiquitination. NRF2 escapes degradation and translocates to the nucleus to activate cytoprotective target genes via antioxidant response elements (AREs). In *KEAP1*-mutant cancers, loss-of-function mutations constitutively mimic this state, rendering NRF2 permanently nuclear.

Under basal, unstressed conditions, NRF2 protein levels are maintained at extremely low levels through continuous proteasomal degradation mediated by its principal repressor, KEAP1 (Figure 2). KEAP1 is a BTB-Kelch protein that functions as a substrate adaptor for the Cullin 3 (CUL3)-RBX1 E3 ubiquitin ligase complex, recruiting NRF2 for polyubiquitination and subsequent degradation by the proteasome (Cullinan et al., 2004; Kobayashi et al., 2004). As a result, NRF2 has a short basal half-life of approximately 15–20 min (Stewart et al., 2003), ensuring that the transcriptional program it regulates remains suppressed in the absence of cellular stress.

KEAP1 exists as a homodimer, with each monomer comprising an N-terminal BTB domain that mediates dimerization and CUL3 binding, a central intervening region (IVR) that harbors key sensor cysteines, and a C-terminal Kelch repeat domain that forms a six-bladed β-propeller responsible for NRF2 binding (Lo et al., 2006). The β-propeller engages the Neh2 domain of NRF2 at two distinct binding sites: the high-affinity ETGE motif, which anchors KEAP1 constitutively (the “hinge”), and the lower-affinity DLG motif, which positions the intervening lysine residues of Neh2 in proximity to the ubiquitin transfer machinery (the “latch”) (McMahon et al., 2006; Tong et al., 2006). Both motifs must engage simultaneously for ubiquitination to occur, and disruption of either contact is sufficient to stabilize NRF2.

The electrophile and oxidant sensing function of KEAP1 is mediated by several reactive cysteine residues located within its BTB and IVR domains, most notably C151, C273, and C288 (Dinkova-Kostova et al., 2002; Zhang and Hannink, 2003). These cysteines act as molecular sensors with distinct roles: C151 is preferentially modified by electrophiles and many chemopreventive compounds, whereas C273 and C288 are required for maintaining basal repression of NRF2 and are particularly sensitive to oxidative stress (Zhang and Hannink, 2003; Dayalan Naidu et al., 2018). Modification of C151 induces conformational changes that weaken KEAP1 binding to the DLG “latch” motif of NRF2 while preserving interaction with the ETGE “hinge.” As a result, KEAP1 remains bound to NRF2 but can no longer efficiently promote its ubiquitination (Wakabayashi et al., 2004; Saito et al., 2015). NRF2 therefore escapes degradation, accumulates, and newly synthesized NRF2 translocate to the nucleus to activate transcription of target genes (Zhang and Hannink, 2003).

NRF2 activity is further regulated by a KEAP1-independent degradation pathway, including GSK-3β- β-TrCP-CUL1 mediated degradation, as described above, as well as by a collection of endogenous protein competitors that disrupt the KEAP1-NRF2 interaction. These include p62/SQSTM1, which binds directly to the BTB domain of KEAP1 to sequester it away from NRF2 during autophagy impairment (Jain et al., 2010; Komatsu et al., 2010; Ichimura et al., 2013); PALB2, which competes with KEAP1 for Neh2 binding (Ma et al., 2012); and WTX, a tumor suppressor that promotes KEAP1-independent NRF2 degradation through interaction with the CUL3 complex (Camp et al., 2012). The existence of these alternative regulatory inputs underscores that NRF2 activity is not exclusively a readout of redox state, but is integrated with autophagy flux, tumor suppressor networks, and developmental signaling, a complexity with important implications for understanding NRF2 dysregulation in cancer.

### 2.3 NRF2 target genes and cellular functions

Upon stabilization and nuclear translocation, NRF2 heterodimerizes with small Maf proteins via its Neh1 domain and binds AREs in the promoters of its target genes, activating several transcriptional programs (Itoh et al., 1997).

The most extensively characterized is the glutathione system. NRF2 directly induces the glutamate-cysteine ligase subunits (*GCLC*, *GCLM*), glutathione synthetase (*GSS*), and glutathione reductase (*GSR*), maximizing both glutathione biosynthesis and recycling capacity (Moinova and Mulcahy, 1999; Wild et al., 1999; Harvey et al., 2009). Cysteine supply for this pathway is secured through NRF2-driven induction of the cystine/glutamate antiporter SLC7A11 (xCT), which imports extracellular cystine in exchange for glutamate (Koppula et al., 2021). Closely coupled to this is NADPH regeneration, achieved through upregulation of pentose phosphate pathway enzymes (*G6PD*, *PGD*) and one-carbon metabolism enzymes (*ME1*, *IDH1*), which collectively replenish the NADPH consumed by glutathione and thioredoxin reductases (*TXNRD1*) (Mitsuishi et al., 2012; Ludtmann et al., 2014).

NRF2 also coordinates xenobiotic detoxification through induction of NQO1 (Jaiswal, 2000), multiple glutathione S-transferase isoforms (Chanas et al., 2002), and aldo-keto reductases (Penning, 2016), enabling cells to neutralize a broad range of electrophilic compounds including chemotherapeutic agents. Iron homeostasis is regulated through *HMOX1* and the ferritin subunits *FTH1* and *FTL*, protecting against iron-catalyzed oxidative damage and conferring resistance to ferroptosis (Alam et al., 1999; Sun et al., 2016). Beyond these canonical functions, NRF2 induces *UGDH* (Doshi et al., 2023; Gebru et al., 2025), linking its transcriptional program to nucleotide sugar biosynthesis, a metabolic consequence with selective vulnerabilities in NRF2-addicted tumors that will be discussed in detail below.

In normal physiology, this program is transiently activated and extinguished as stress resolves. In cancer, its constitutive activation drives cancer cell survival and proliferation.

## 3 NRF2 in cancer: from cytoprotector to oncogenic driver

### 3.1 Frequency and mechanisms of NRF2 pathway activation in cancer

While NRF2’s cytoprotective role is essential in normal physiology, its constitutive activation is a hallmark of many cancers (Sporn and Liby, 2012). Mutations disrupting the KEAP1-NRF2 regulatory axis are among the most frequently detected alterations in human malignancy, occurring through several distinct mechanisms. Loss-of-function mutations in *KEAP1* are the most common, occurring in approximately 20%–30% of lung adenocarcinomas (Collisson et al., 2014) and at lower but significant frequencies in hepatocellular carcinoma, head and neck squamous cell carcinoma, and bladder cancer (Kim et al., 2010; Haque et al., 2020; Wei et al., 2024). Gain-of-function mutations in *NFE2L2* itself represent a second mechanism, clustering almost exclusively at the DLG and ETGE motifs of the Neh2 domain to directly disrupt KEAP1 binding and render NRF2 constitutively active (Shibata et al., 2008; Goldstein et al., 2016). Although at lower frequencies, the CUL3 E3 ligase is also mutated in some lung cancers (Jin et al., 2021), leading to loss of NRF2 ubiquitination and degradation. Mutations in KEAP1, NRF2 and CUL3 are mutually exclusive, suggesting they all achieve the same end (Jin et al., 2021). Additional mechanisms of pathway activation include epigenetic silencing of *KEAP1* by promoter hypermethylation (Wang et al., 2008; Camiña and Penning, 2022), accumulation of p62/SQSTM1 through autophagy impairment (Ichimura et al., 2013), and oncogene-driven transcriptional upregulation of *NFE2L2* downstream of KRAS and MYC activation (Denicola et al., 2011; Taguchi and Yamamoto, 2020). Elevated succinate as a result of inactivating mutations in fumarate hydratase can also result in chronic inactivation of KEAP1, by electrophilic adduction of reactive cysteines in KEAP1 (Adam et al., 2011). The functional consequence of all these different mechanisms is constitutive NRF2 nuclear localization leading to increased transcription of its target genes ultimately allowing the tumor to acquire broad stress and drug resistance capabilities.

### 3.2 Metabolic reprogramming

One of the oncogenic consequences of constitutive NRF2 activation is reprogramming of cellular metabolism toward anabolic biosynthesis. NRF2 diverts glucose flux into the pentose phosphate pathway, simultaneously generating NADPH for antioxidant defense and ribose-5-phosphate for nucleotide biosynthesis. Glutamine metabolism is similarly remodeled: NRF2 promotes glutamine utilization not only for glutathione biosynthesis via NRF2-driven induction of GCLC, GCLM, and SLC7A11, which continuously exports glutamate in exchange for cystine, but also for anaplerotic TCA cycle entry, providing carbon for lipid synthesis and energy production under conditions of reduced glycolytic flux (Romero et al., 2017). NRF2 upregulates serine biosynthesis, one-carbon metabolism and purine biosynthesis enzymes including PPAT, expanding the nucleotide pools required for rapid proliferation (DeNicola et al., 2015; Okazaki et al., 2020). NRF2-driven induction of *UGDH* channels UDP-glucose into UDP-glucuronic acid production, a metabolic flux that, as will be discussed, creates a selective vulnerability to UXS1 loss in *KEAP1*-mutant tumors (Doshi et al., 2023; Gebru et al., 2025).

At the intersection of metabolism and epigenetics, NRF2 has been implicated in supporting acetyl-CoA availability through regulation of citrate export and ATP-citrate lyase activity, potentially linking its transcriptional program to histone acetylation and epigenetic state (Hayes and Dinkova-Kostova, 2014). Finally, NRF2 promotes lipid metabolic remodeling through upregulation of fatty acid synthesis enzymes and stearoyl-CoA desaturase, supporting membrane biogenesis and providing an additional layer of ferroptosis resistance (Koppula et al., 2021; Chen et al., 2024). Together, these interconnected metabolic shifts reflect a coordinated reorientation of central carbon metabolism that positions NRF2 not merely as a redox regulator but as a master coordinator of the biosynthetic and energetic demands of constitutively stressed, rapidly proliferating cancer cells.

### 3.3 Drug resistance

Constitutive NRF2 activation is one of the most potent and broadly acting mechanisms of cancer drug resistance. By maintaining high levels of glutathione and inducing several phase II detoxifying enzymes, NRF2 directly inactivates electrophilic, chemotherapeutic and alkylating agents, and anthracyclines (Tew, 2016). Beyond direct drug inactivation, NRF2 confers resistance to radiation through enhanced ROS scavenging, and to targeted therapies through transcriptional upregulation of compensatory survival pathways (Singh et al., 2010; Jeddi et al., 2017; Jeong et al., 2017; Hellyer et al., 2019). Additionally, NRF2 drives expression of multidrug resistance transporters including MRP1-5 (encoded by *ABCC* family genes), promoting efflux of chemotherapeutic agents before they can reach their intracellular targets (Hayashi et al., 2003). Because of this, *KEAP1/NFE2L2* mutations are consistently associated with poor prognosis and reduced clinical response to chemotherapy across different tumor types (Nadal et al., 2019; Scalera et al., 2022; Zavitsanou et al., 2023; Li et al., 2024). KEAP1/NRF2 mutations are also associated with poor outcomes following immunotherapy. This is associated with low tumor immune cell infiltration, increased PD-L1 expression and decreased dendritic and T cells responses (Zavitsanou et al., 2023; Li et al., 2024) These findings underscore the clinical urgency of developing effective strategies to overcome NRF2-driven resistance to tumor intrinsic and immune mediated therapies.

### 3.4 Ferroptosis resistance

Another important consequence of NRF2 activation in cancer is resistance to ferroptosis, a form of regulated cell death driven by iron-dependent lipid peroxidation (Li et al., 2020; Chen et al., 2024). NRF2 suppresses ferroptosis through multiple transcriptional mechanisms. Induction of *SLC7A11* sustains cystine import and glutathione synthesis, preserving GPX4 activity (Koppula et al., 2021). Upregulation of HMOX1 and ferritin subunits limits the accumulation of labile iron (Kerins and Ooi, 2018). NRF2 also enhances the CoQ–FSP1 antioxidant system through increased expression of FSP1 (AIFM2) (Emmanuel et al., 2022; Koppula et al., 2022), providing a glutathione-independent mechanism for detoxifying lipid peroxides. The clinical relevance of this is significant as ferroptosis has emerged as a promising therapeutic modality in cancers resistant to apoptosis-inducing agents, and *KEAP1* mutations represent one of the major causes of ferroptosis resistance that must be overcome to realize this therapeutic potential.

### 3.5 Tumor microenvironment remodeling

Constitutive NRF2 activation can also shape the tumor microenvironment to promote immune evasion and therapeutic resistance. NRF2 suppresses pro-inflammatory cytokine production, including IL-1β, IL-6, and TNFα by interfering with NF-κB signaling, reducing the inflammatory state of the tumor microenvironment (Thimmulappa et al., 2006; Kobayashi et al., 2016). *KEAP1*-mutant lung adenocarcinomas are characterized by an immune-cold microenvironment with reduced cytotoxic T cell infiltration and diminished response to immune checkpoint blockade, consistent with NRF2-mediated suppression of immunogenic signaling (Best et al., 2019; Okazaki et al., 2020). The mechanisms underlying this immune evasion remain incompletely understood but likely involve NRF2-driven suppression of antigen presentation, reduction of damage-associated molecular pattern signaling, and metabolic competition within the tumor microenvironment. These features reduce the efficacy of immunotherapy making the immune-cold phenotype of NRF2-addicted tumors an important and underexplored therapeutic challenge.

## 4 Targeting NRF2 in cancer: current inhibitor strategies and their limitations

### 4.1 Current pharmacological approaches to NRF2 inhibition

Given the frequencies of NRF2 activation in human tumors, and the poor prognosis for these patients, identification of NRF2 inhibitors has become a high priority for cancer researchers. Cell-based compound screening approaches using cells expressing a reporter gene (such as luciferase) driven by one or more copies of antioxidant response elements (AREs) that drive the expression of NRF2 target genes, are a popular and convenient approach to screen for potential NRF2 inhibitors. Although this approach is fast and high throughput, it is prone to several assay artifacts, including lack of specificity, identification of toxic compounds, cell line specific artifacts, and lack of appropriate cellular context. Hits from such screens therefore require rigorous followup to address these and other limitations. Screening a large number of natural products using an ARE reporter in MDA-MB-231 cells led to the identification of the quassinoid brusatol (Ren et al., 2011). This compound inhibited the expression of NRF2 and its target genes at low concentrations and reduced the proliferation and tumor growth of the *KEAP1* mutant Non-small Cell Lung Cancer (NSCLC) cell line, A549. Many publications have used brusatol to draw conclusions about the specific effects of NRF2 inhibition *in-vitro* and *in-vivo*. However, caution should be applied to these interpretations as brusatol has been known for many years to act as a general protein synthesis inhibitor and at concentrations and time points used to decrease NRF2 levels, many additional short-lived proteins are also lost (Vartanian et al., 2016; Harder et al., 2017).

A similar ARE-driven reporter gene assay in A549 cells screened against 5,861 compounds led to the identification of febrifugine, a plant alkaloid (Tsuchida et al., 2017). A synthetic derivative, halofuginone, was shown to decrease NRF2 proteins levels in *KEAP1* mutated cells without affecting NRF2 mRNA levels. Halofuginone is also a general protein synthesis inhibitor, due, at least in part, to its ability to inhibit prolyl tRNA synthetase and activate the eIF2a kinase GCN2. The authors confirmed that halofuginone indeed acts through this mechanism, as NRF2 levels were rescued upon proline supplementation (Tsuchida et al., 2017).

Another ARE-reporter screen of ∼400,000 small molecules in A549 cells identified ML385, which acts by binding the Neh1 domain of NRF2 and inhibiting association of the NRF2/MafG complex to DNA (Singh et al., 2016). This compound decreases NRF2 activity in *KEAP1* mutant cells and decreases colony numbers alone and in combination with chemotherapeutic drugs. It also decreases tumor growth in combination with carboplatin in sub-cutaneous and lung orthotopic models. Several studies have demonstrated potential therapeutic applications of ML385 *in-vitro* and *in-vivo*, but low potency, ambiguous specificity and poor pharmacological properties have impeded progression to clinical trials.

More recently, while optimizing covalent inhibitors of KEAP1 for autoimmune indications, Vividion discovered a series of KEAP1 C151 reactive molecules that allosterically activate, rather than inhibit KEAP1 (Roy et al., 2025). >1,000 covalent compounds were screened in cells for the ability to engage KEAP1 C151, and one series was identified that unexpectedly reduced NRF2 levels and transcriptional output. This series, exemplified by VVD-065, is quite selective for KEAP1 engagement, and stabilizes a conformation of KEAP1 that favors binding to CUL3, which results in increased ubiquitination and proteasome mediated degradation of NRF2. VVD-065 reduces viability in some KEAP1 mutant models in 3D-sphere and *in-vivo* environments. Importantly, the mechanism of action of VVD-065 requires sufficient expression of KEAP1, and mutations that maintain sufficient basal interaction between KEAP1 and NRF2. Therefore, strong decreases in KEAP1 protein through genomic loss or promoter methylation, as well as KEAP1 or NRF2 mutations that completely abolish protein interaction, will likely be insensitive to these compounds. Nevertheless, a related compound VVD-037 is currently in clinical trials in patients with advanced solid tumors.

Recently, Donovan et al. explored the possibility of repurposing compounds that mediate degradation of β-catenin by β-TRCP to also degrade Nrf2. β -TRCP is an E3 ligase that regulates NRF2 ubiquitination under certain cellular conditions (Rada et al., 2011). While these and related compounds could promote binding of Nrf2 degron peptides to β-TRCP *in-vitro*, they did not show any ability to cause Nrf2 degradation in cells, possibly due to occlusion of the compound binding site in ligand free Nrf2/β-TRCP complexes (O Donovan et al., 2026). Nevertheless, an inventive two step screen of 125,000 compounds showing selective viability effects in KEAP1 mutant cells followed by analysis of effects on NRF2 target gene expression identified ARP-4922, which binds and degrades NRF2 in a manner that involves recruitment of β-TRCP. ARP-4922 showed impressive anti-tumor activity in KEAP1 and NRF2 mutant lung cancer xenograft models (presented at AACR general meeting, 2025 (Read et al., 2025).

### 4.2 Why direct NRF2 inhibition remains elusive

The pharmacological trajectory outlined above reflects the difficulty of translating a compelling oncological target into an effective clinical therapy, owing to both structural and biological constraints.

The primary challenge is structural undruggability. Unlike kinases or proteases, NRF2 exerts its oncogenic function through large, flat protein-protein and protein-DNA interaction surfaces that lack well-defined binding pockets (Cloer et al., 2019; Telkoparan-Akillilar et al., 2021). The ARE-based reporter screens that identified brusatol and halofuginone are inherently prone to the problem of selecting for any compound that reduces NRF2-dependent transcription regardless of mechanism, and, as demonstrated for both agents, identifying general translation inhibitors whose apparent NRF2 selectivity is an artifact of NRF2’s short half-life.

The allosteric KEAP1 activators represented by VVD-065 circumvent this problem elegantly but introduce a patient selection constraint. Efficacy requires residual KEAP1 protein and a preserved KEAP1-NRF2 interaction interface. The substantial proportion of KEAP1-mutant NSCLC tumors in which KEAP1 is absent through genomic deletion, promoter methylation, or truncating mutations will be intrinsically resistant, narrowing the population with tumor type where NRF2 addiction is most prevalent.

Beyond druggability, two biological challenges compound the difficulty. First, systemic NRF2 suppression carries meaningful normal tissue toxicity risk. NRF2 is non-redundantly required for cytoprotection in the lung, kidney, liver, and brain, and cancer patients undergoing cytotoxic therapy that induce oxidative stress are the population least able to tolerate its loss. Second, NRF2 is tumor suppressive in the pre-malignant setting and becomes oncogenic only in established KEAP1/NFE2L2-mutant tumors and a blunt inhibitor risks suppressing protective NRF2 in normal tissue while targeting oncogenic NRF2 in tumor cells, an unfavorable therapeutic index that tumor-selective delivery can potentially resolve. Together, these challenges define the conceptual space that has motivated the synthetic lethality framework developed in the following section.

An alternative to direct NRF2 inhibition is to target druggable proteins within the NRF2 transcriptional network that are selectively expressed in *KEAP1*-mutant tumors. Bar-Peled et al. demonstrated this concept using chemical proteomics to map cysteine ligandability across *KEAP1*-mutant and *KEAP1*-WT NSCLC cell lines (Bar-Peled et al., 2017). They identified NR0B1, an orphan nuclear receptor whose expression is tightly linked to *KEAP1/NRF2* mutational status, as a top druggable hit. NR0B1 is in a transcriptional complex with RBM45 and SNW1 that co-regulates approximately half of the NRF2 gene expression program involved in pro-proliferative pathways. They identified a cysteine residue within the NR0B1 protein-interaction domain (C274) that is amenable to covalent targeting. Small-molecule ligands directed at this site disrupted NR0B1-containing complexes and selectively impaired anchorage-independent growth in KEAP1-mutant cells, with minimal effects in KEAP1-WT cells. Although their tool compounds had a modest cellular potency and require further optimization before clinical translation, these findings establish a broader principle that NRF2-regulated proteins can serve as surrogates for NRF2 inhibition, circumventing its structural undruggability, which is the focus of the following section.

## 5 Exploiting NRF2 addiction: synthetic lethality as an alternative therapeutic strategy

**FIGURE 3:**
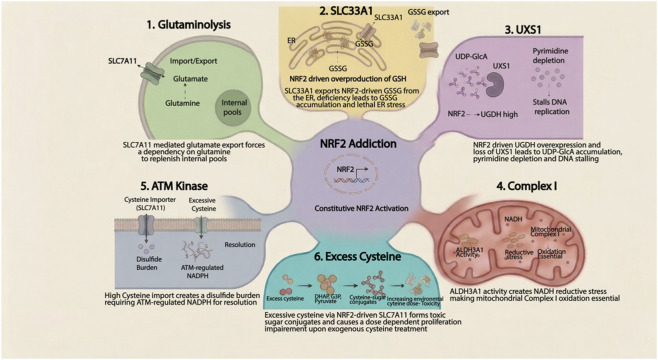
NRF2 addiction-driven synthetic lethal vulnerabilities in *KEAP1*-mutant cancer. Constitutive NRF2 activation creates six therapeutically exploitable dependencies. (1) Glutaminolysis: SLC7A11-mediated glutamate export drives dependency on glutaminolysis to replenish intracellular glutamate. (2) SLC33A1 and ER redox: NRF2-driven glutathione overproduction elevates ER GSSG; SLC33A1 loss blocks GSSG export, causing lethal ER hyperoxidation. (3) UXS1: NRF2-driven UGDH overexpression elevates UDP-GlcA; UXS1 loss causes pyrimidine depletion and replication fork stalling. (4) Complex I: NRF2-driven ALDH3A1 activity generates NADH reductive stress, creating dependency on mitochondrial Complex I for NADH oxidation. (5) ATM kinase: Constitutively elevated cystine import creates a disulfide burden requiring ATM-regulated NADPH for resolution; ATM inhibition causes selective cell death. (6) Excess cysteine: Surplus intracellular cysteine reacts with glycolytic intermediates to form toxic cysteine-sugar conjugates, impairing proliferation in a dose-dependent manner. In each case, selectivity derives from NRF2’s own transcriptional program.

The challenges of directly inhibiting NRF2 have prompted a shift in therapeutic strategy. In tumors harboring mutations in KEAP1, where NRF2 is constitutively nuclear and its transcriptional program persistently active, this state generates genotype-specific vulnerabilities that can be therapeutically targeted without directly inhibiting NRF2 and sparing normal tissues. Consistent with this concept, multiple studies have identified synthetic lethal interactions in NRF2-addicted tumors (Figure 3).

### 5.1 Glutaminolysis dependency

The first and most clinically advanced synthetic lethal vulnerability in *KEAP1*-mutant cancer is dependency on glutaminolysis. In *KEAP1*-mutant cancer cells, the constitutive activation of NRF2 induces a state of “glutamine addiction” that creates a prime opportunity for synthetic lethal intervention. This dependency primarily stems from the significant upregulation of the cystine/glutamate antiporter SLC7A11 (xCT), which is a transcriptional target of NRF2. To maintain the high intracellular levels of glutathione necessary for redox defense, NRF2-addicted cells continuously import cystine in exchange for exporting glutamate. To sustain this high-volume export and prevent glutamate depletion, NRF2-addicted cells become dependent on glutaminolysis (the enzymatic conversion of glutamine to glutamate) to replenish the internal pool (Mitsuishi et al., 2012). This dependency was validated in genetically engineered mouse models as well as human cancer cell lines (Romero et al., 2017). Increased PI3K/Akt signaling was shown to cooperate with KEAP1 mutations resulting in increased NRF2 nuclear localization, target gene expression and metabolic function. This might contribute to the co-occurrence of KEAP1 and KRAS and KEAP1 and PIK3CA mutations found in lung and Head and Neck cancers. Based on this data, the glutaminase inhibitor telaglenastat (CB-839) was evaluated in the phase II KEAPSAKE trial in combination with pembrolizumab and chemotherapy as first-line therapy for KEAP1/NRF2-mutated non-squamous metastatic NSCLC (Skoulidis et al., 2020) but was discontinued following a negative result in 2021. The trial’s failure underscored that blocking a single glutamine-consuming reaction may be insufficient given the level of NRF2-driven glutamine dependencies. A subsequent phase I trial is currently evaluating telaglenastat with the dual mTORC1/2 inhibitor sapanisertib (Riess et al., 2020), based on the rationale that dual blockade of glutaminolysis and glycolysis is required to overcome the metabolic plasticity of NRF2-addicted tumors (Shibata et al., 2010).

The KEAPSAKE trial experience also prompted interest in pan-glutamine antagonism. The tumor-targeted DON prodrug DRP-104 (Rais et al., 2022)suppresses KEAP1-mutant tumor growth by inhibiting multiple glutamine-dependent enzymes and blocking glutamine flux across nucleotide synthesis and other downstream pathways that telaglenastat does not target (Pillai et al., 2024). Furthermore, selectivity of DRP-104 is enhanced by NRF2-driven induction of CES1, an esterase that mediates intratumoral prodrug activation. In addition to metabolic cytotoxicity, DRP-104 has been shown to reverse T cell exhaustion and enhance CD4^+^ and CD8^+^ effector function, synergizing with anti-PD-1 therapy in orthotopic *KEAP1*-mutant models (Pillai et al., 2024). Together, these findings highlight glutamine antagonism as a strategy that simultaneously exploits a metabolic dependency and counteracts the immune-cold phenotype of NRF2-addicted tumors.

### 5.2 SLC33A1 and ER proteostatic stress

The glutaminolysis results prompted an unbiased screening effort to identify additional *KEAP1*-mutant dependencies beyond the glutamine pathway. Romero et al. performed a druggable genome CRISPR-Cas9 screen in isogenic *Keap1*-mutant murine lung adenocarcinoma (LUAD) cell lines derived from KP (*Kras*-mutant, *Trp53*-/-) and KPK (*Kras*-mutant, *Trp53*-/-, *Keap1*-/-) models. Their analysis identified SLC33A1, an ER-resident protein as the top *Keap1*-mutant-specific dependency, along with several genes related to the unfolded protein response (UPR), including SUCO and TAPT1, all of which were cross-validated against the publicly available DepMap dependency data. Validation in multiple independent *Keap1*-mutant mouse and human cancer cell lines, and in preclinical KPK genetically engineered mouse models (GEMMs), confirmed SLC33A1 loss as selectively lethal in the *KEAP1*-mutant context, with attenuated effects in isogenic *KEAP1* wild-type cells (Romero et al., 2020).

The mechanistic basis of this dependency has been substantially revised by two recent studies. SLC33A1 was originally proposed to transport acetyl-CoA into the ER to support protein acetylation and autophagy, with the dependency attributed to impaired proteostatic clearance under the transcriptional burden of constitutive NRF2 activation. Liu et al. now demonstrate, using ER immunopurification, CRISPR screening, liposome reconstitution, and cryo-EM, that SLC33A1 is in fact the major GSSG exporter of the mammalian ER. In *KEAP1*-mutant cells, NRF2-driven glutathione overproduction increases oxidative protein folding activity and GSSG generation within the ER (Liu et al., 2026). SLC33A1 exports this GSSG to the cytosol for recycling; without it, GSSG accumulates in the ER lumen, hyperoxidizing protein disulfide isomerases and triggering ER stress and ERAD dependency. The selective vulnerability of *KEAP1*-mutant cells therefore reflects a specific imbalance in ER redox homeostasis proportionate to their constitutively elevated glutathione pool, rather than a generalized proteostatic burden (Liu et al., 2026).

Kutseikin et al. extended this mechanistic framework by identifying IXA4 as the first pharmacological SLC33A1 inhibitor (Kutseikin et al., 2026). Cryo-EM and chemoproteomic target engagement studies showed that IXA4 binds the central channel of SLC33A1 to block metabolite transport, causing ER GSSG accumulation and lumen hyperoxidation. IXA4 selectively reduced viability of KPK but not KP cells, an effect abolished by BSO-mediated glutathione depletion, directly confirming that lethality depends on the elevated GSH pool characteristic of *KEAP1*-deficient cells. Critically, IRE1 RNase inhibition did not rescue KPK cell death, establishing that therapeutic efficacy is driven by ER redox disruption rather than downstream UPR signaling. IXA4 represents the first tool compound to pharmacologically validate the SLC33A1 vulnerability, and the finding that NRF2 transcriptional activity score predicts dependency more robustly than *KEAP1* mutation status alone suggests the addressable population may extend beyond canonically *KEAP1*-mutant tumors.

### 5.3 UXS1 dependency

Another example of NRF2 addiction-derived vulnerability is the selective dependency of *KEAP1*-mutant tumors on UDP-xylose synthase 1 (UXS1), recently described by our group (Gebru et al., 2025) as well as others (Doshi et al., 2023). NRF2 drives high expression of UDP-glucose 6-dehydrogenase (UGDH), which converts UDP-glucose to UDP-glucuronic acid (UDP-GlcA). NRF2-driven high UGDH expression in *KEAP1*-mutant cells leads to increased UDP-GlcA synthesis that requires functional UXS1 to convert it to UDP-xylose; UDP-xylose in turn acts as a negative feedback regulator of UGDH, forming a homeostatic loop that is disrupted when UXS1 is lost. In *KEAP1*-mutant cells, UXS1 loss causes UDP-GlcA to accumulate to a degree that sequesters available UDP, depleting the cell’s pyrimidine nucleotide pool. The resulting shortage of available UDP and other pyrimidines slows S-phase progression and stalls DNA replication forks, causing cells to undergo prolonged cell-cycle exit and/or apoptosis selectively in *KEAP1*-mutant tumors. The dependency on UXS1 was rescued by *UGDH* knockout confirming that it is the NRF2-driven overexpression of UGDH, and not UXS1 loss induced inhibition of proteoglycan synthesis, that creates the vulnerability. DNA replication stress in UXS1-knockout cells sensitized them to clinical cell-cycle checkpoint inhibitors, opening an additional combination window. Importantly, these effects were selective to *KEAP1*-mutant cells, with *KEAP1* wild-type cells unaffected by UXS1 loss and despite the liver having the highest normal tissue expression of UGDH, UXS1 knockout in the liver did not produce hepatotoxicity, providing an early indication of a favorable therapeutic window.

Notably, Doshi et al. also independently demonstrated that UXS1 loss in high-UGDH-expressing cells causes UDP-GlcA accumulation and cell death. Their study demonstrates a distinct mechanism of cell death. They showed that UDP-GlcA toxicity stems primarily through disruption of Golgi morphology and function, impeding trafficking of surface proteins, including EGFR, to the plasma membrane and attenuating downstream signaling. Therefore, the relative contribution of Golgi disruption versus pyrimidine depletion may be determined by the absolute magnitude of UDP-GlcA accumulation and the metabolic state of the cell.

### 5.4 NADH reductive stress and complex I inhibition

A counterintuitive vulnerability was identified by Weiss-Sadan et al., who systematically induced NRF2 activation across a panel of NSCLC cell lines using the KEAP1 inhibitor KI696 and observed that rather than uniformly promoting proliferation, acute NRF2 activation paradoxically blocked growth in over 13% of lines tested, an effect fully rescued by NRF2 ablation (Weiss-Sadan et al., 2023). The mechanism involves NRF2-driven upregulation of ALDH3A1, an NAD^+^-consuming aldehyde dehydrogenase whose activity generates NADH as a byproduct. When ALDH3A1 induction tips the NADH/NAD^+^ ratio beyond a critical threshold, cells enter a state of reductive stress that blocks proliferation.

The identity of the sensitive cell lines was mechanistically informative. Sensitivity to KEAP1 inhibitor-induced reductive stress was restricted to *KEAP1* wild-type cells with low baseline glycolytic flux. In *KEAP1* wild-type cells that have robust glycolysis, lactate dehydrogenase efficiently regenerates NAD^+^, neutralizing the additional NADH burden imposed by high ALDH3A1. *KEAP1*-mutant cells with constitutive NRF2 activity chronically direct glucose flux into the pentose phosphate pathway, generating NADPH rather than regenerating NAD^+^ (Mitsuishi et al., 2012). While *KEAP1*-mutant cells have adapted to tolerate this chronic state, this adaptation comes at the cost of reliance on mitochondrial Complex I as the primary route of NADH oxidation. Hence, Complex I inhibition with agents, such as IACS-010759 or potentially metformin, overwhelms this residual capacity, pushing *KEAP1*-mutant cells into lethal reductive stress. This study illustrates that therapeutic selectivity in this context derives not from a single target gene but from the low-glycolytic metabolic state that NRF2 addiction causes in susceptible *KEAP1*-mutant cells, a vulnerability shared by a subset of low-glycolytic *KEAP1* wild-type cells.

### 5.5 ATM kinase synthetic lethality

A metabolism-centered CRISPR screen designed to identify metabolic pathways conferring resistance to ATM kinase inhibition unexpectedly implicated KEAP1 as the top sensitizing hit across breast and lung cancer models (Li et al., 2023) KEAP1 loss drives constitutive NRF2 activation and high SLC7A11 expression, creating chronic cystine dependency. Elevated cystine import increases the intracellular disulfide burden, which must be continuously resolved by NADPH-dependent thioredoxin and glutathione systems. ATM inhibition depletes cellular NADPH through its underappreciated role in pentose phosphate pathway regulation, impairing this disulfide resolution capacity. In *KEAP1*-deficient cells, where the disulfide burden is already constitutively elevated, ATM inhibition tips the redox balance into unresolvable disulfide stress and selective cell death, a mechanism confirmed by rescue with SLC7A11 inhibition or the reducing agent N-acetylcysteine, directly implicating disulfide stress rather than DNA damage as the proximal lethal event.

*In vivo*, the clinical-stage ATM inhibitor AZD1390 suppressed growth of KEAP1-deficient breast and lung xenografts, highlighting a near-term translational opportunity. However, evidence to date is largely limited to xenograft models, and the impact of ATM inhibition on the immune-cold microenvironment characteristic of KEAP1-mutant tumors remains to be defined.

### 5.6 Excess cysteine and SLC7A11-driven vulnerability

A recent study by Brain et al. identified a further consequence of constitutive NRF2 activation (Brain et al., 2026). The authors used an unbiased isotope-tracing approach to systematically catalogue the metabolic fates of cysteine in NRF2-activated cancer cells. NRF2-driven SLC7A11 overexpression drives cystine import far in excess of the cellular demands of glutathione synthesis and protein incorporation. They identified a class of previously uncharacterized cysteine-sugar conjugates formed by spontaneous reactions between surplus intracellular cysteine and glycolytic intermediates including DHAP, G3P, and pyruvate that accumulate selectively in NRF2-activated cells. Two conjugates, 3GC and 1DC, were confirmed in autochthonous NRF2-mutant murine LUAD models and in primary human squamous cell lung cancer samples carrying *KEAP1/NFE2L2* mutations, establishing their *in-vivo* and clinical relevance as biomarkers of NRF2 pathway addiction.

Functionally, excess intracellular cysteine is itself toxic and NRF2-activated cells with high SLC7A11 expression showed dose-dependent proliferation impairment at elevated environmental cystine concentrations, which is rescued by xCT inhibition and absent in SLC7A11-low cells. Together with the ATM synthetic lethality work, this study reinforces that the SLC7A11/cystine axis represents a nexus of multiple independent therapeutic vulnerabilities in *KEAP1*-mutant tumors, each exploitable through mechanistically orthogonal approaches.

## 6 Conclusion

In the three decades since its discovery, NRF2 has evolved from an erythroid transcription factor of uncertain significance into a central regulator of cancer biology. This trajectory reflects a fundamental duality: in normal tissues, NRF2 preserves cellular homeostasis by coordinating responses to oxidative and electrophilic stress, whereas in cancer, this same program is co-opted by mutations in KEAP1 and NFE2L2 to drive constitutive transcriptional activation, metabolic rewiring, therapy resistance, ferroptosis evasion, and immune exclusion, hallmarks of some of the most refractory tumors in oncology.

Direct inhibition of NRF2, while conceptually appealing, is limited by its structural undruggability as a transcription factor, the risk of systemic toxicity, and context-dependent biology that complicates patient selection. Despite substantial investment, including the recent entry of the first allosteric KEAP1 activator VVD-037 into clinical trials, no NRF2-targeted therapy has yet achieved clinical approval in oncology. These challenges include the mechanistic non-selectivity of earlier tool compounds, the patient population constraints of *KEAP1*-dependent approaches, and the dual-role paradox that makes NRF2 simultaneously tumor suppressive in normal tissue and oncogenic in established tumors.

The alternative framework advanced in this review, exploiting the metabolic vulnerabilities imposed by NRF2 addiction rather than suppressing NRF2 itself offers a conceptually more tractable path. The evidence now spans multiple independent metabolic axes: glutaminolysis, ER proteostatic stress through the GSSG export function of SLC33A1, NADH reductive stress, cystine metabolism and ATM synthetic lethality, nucleotide sugar biosynthesis through the UGDH/UXS1 axis, and excess cysteine stress. In each case, the vulnerability is mechanistically rooted in NRF2’s own transcriptional program, and in each case selectivity for *KEAP1*-mutant or NRF2-hyperactivated cells over normal tissues has been demonstrated preclinically.

Realizing this potential will require advances in several areas, such as systematically mapping the full landscape of NRF2-dependent vulnerabilities and developing rational combination therapies to overcome metabolic plasticity. It is important to note, that the synthetic lethal vulnerabilities reviewed here have to date been validated predominantly in preclinical models, and their clinical tractability remains to be fully established. Tumor lineage, metabolic microenvironment, immune context, and co-mutation status are all likely to influence therapeutic response in ways that cannot yet be fully anticipated from preclinical data alone. Whether the compelling mechanistic case reviewed here can ultimately be converted into meaningful clinical benefit will depend on how effectively the field addresses these scientific and translational challenges and on whether patients with NRF2-addicted tumors, who have long faced poor outcomes, can be matched to therapies that exploit the biology that defines their disease.
